# Green Extraction of Carotenoids from Fruit and Vegetable Byproducts: A Review

**DOI:** 10.3390/molecules27020518

**Published:** 2022-01-14

**Authors:** Ewelina Kultys, Marcin Andrzej Kurek

**Affiliations:** Department of Technique and Food Development, Institute of Human Nutrition Sciences, Warsaw University of Life Sciences (WULS-SGGW), Nowoursynowska 159 c Street, 02-776 Warsaw, Poland; Ewelina_Kultys@sggw.edu.pl

**Keywords:** green extraction methods, carotenoids, waste utilization, byproducts, edible oil

## Abstract

Carotenoids are characterized by a wide range of health-promoting properties. For example, they support the immune system and wound healing process and protect against UV radiation’s harmful effects. Therefore, they are used in the food industry and cosmetics, animal feed, and pharmaceuticals. The main sources of carotenoids are the edible and non-edible parts of fruit and vegetables. Therefore, the extraction of bioactive substances from the by-products of vegetable and fruit processing can greatly reduce food waste. This article describes the latest methods for the extraction of carotenoids from fruit and vegetable byproducts, such as solvent-free extraction—which avoids the costs and risks associated with the use of petrochemical solvents, reduces the impact on the external environment, and additionally increases the purity of the extract—or green extraction using ultrasound and microwaves, which enables a significant improvement in process efficiency and reduction in extraction time. Another method is supercritical extraction with CO_2_, an ideal supercritical fluid that is non-toxic, inexpensive, readily available, and easily removable from the product, with a high penetration capacity.

## 1. Introduction

**Figure 1:**
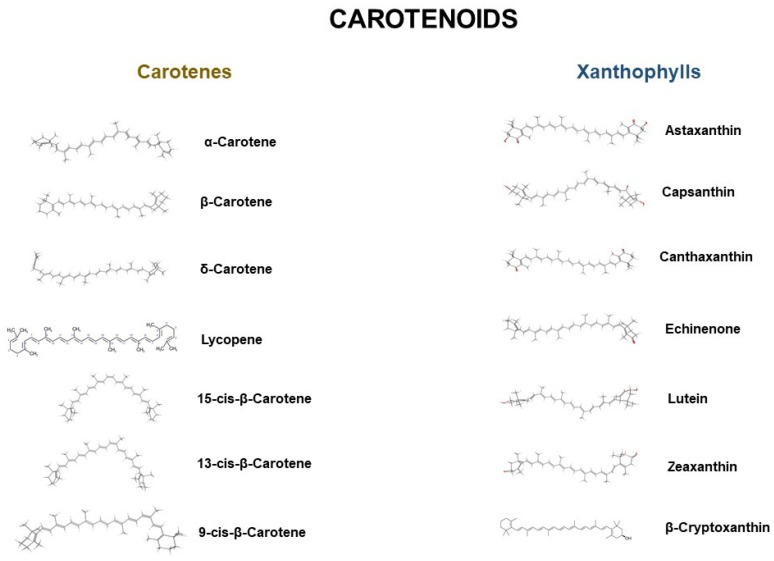
Representative chemical structures of carotenoids.

Carotenoids belong to a group of organic chemical compounds. They are naturally occurring pigments in plants, fungi, algae, and bacteria. They are mainly composed of 40-carbon terpenoids with 8 isoprenoid units as the basic structural unit [1]. More than 650 described carotenoids are found in nature, and can be divided into 2 groups. The first group consists of carotenes, composed only of a hydrocarbon chain without any functional groups, such as lycopene and beta-carotene. The second group consists of xanthophylls, which contain oxygen in their chain in the functional group (e.g., alcohols, aldehydes, ketones); this group includes, e.g., lutein and lute zeaxanthin. In addition, we also distinguish between hydrophobic and hydrophilic carotenoids (Figure 1). Depending on the polarity, different solvents are used in the extraction process. For non-polar carotenoids, the most commonly used solvents are hexane, petroleum ether, and tetrahydrofuran. For polar carotenoids, on the other hand, acetone, ethanol, or ethyl acetate are most commonly used [2]. Standard extraction methods threaten the external environment due to the use of toxic petrochemical solvents. At the same time, the growing interest in carotenoids, due to their health-promoting properties and their potential use in industries as natural pigments, promotes the development of the carotenoids market. The article presents the latest green methods of carotenoid extraction from byproducts of vegetable and fruit processing.

### 1.1. Characteristics of Carotenoids

Carotenoids are classified as fat-soluble micronutrients, characterized by a broad spectrum of health-promoting properties. An increased carotenoid content in the daily diet may reduce the risk of chronic conditions, such as cancer or coronary heart disease [3]. Carotenoids have strong antioxidant, anti-inflammatory and anticancer effects (preventing lung, breast, prostate, colorectal and ovarian cancer). Carotenoids have been proven to have a protective effect on cardiovascular disease. The consumption of processed tomato products contributes to lowering the susceptibility of lipoproteins to oxidative damage, thus preventing hypertension and atherosclerosis. Furthermore, carotenoids help to reduce oxidative stress, which contributes to osteoporosis. The relationship of lycopene in blood serum with the risk of the disease has also been investigated. It was found that its presence is directly related to a reduced risk of osteoporosis. In addition, carotenoids play a key role in strengthening the immune system. Studies have shown that the daily consumption of beta-carotene helps to improve the activity of natural killer (NK) cells [3]. Moreover, beta-carotene is a precursor of vitamin A, whose deficiency in preschool children and pregnant women can result in blindness, poor growth, or even death. Vitamin A is essential for maintaining normal vision and the prevention of eye diseases. Of particular importance in this regard are two carotenoids: lutein and zeaxanthin, whose effects have been documented in the prevention of macular degeneration (AMD), the leading cause of vision loss in people over 65 years of age [4]. Additionally, some of the carotenoids consumed with the daily diet accumulate in the skin and effectively protect it from harmful UV radiation, such as damage, burns, and skin aging [4]. Unfortunately, excess carotenoids can also be harmful. Too much lycopene in the diet can cause an orange discoloration on the skin. This phenomenon is referred to as lycopenodermia. A similar phenomenon is carotenoderma, which manifests as yellow spots on the skin and is caused by excessive levels of carotenoids in plasma. This condition can be achieved by a daily intake of more than 30 mg of beta-carotene over a long period of time. Other adverse risks from excess carotenoids in the diet are reproductive disorders, leukopenia, allergic reactions and increased risk of prostate cancer [3].

Humans cannot synthesize carotenoids themselves and must take them in through food. The main dietary sources of carotenoids are carrots, sweet peppers, and pumpkins. No fewer carotenoids are also found in grape leaves, chili, raw sweet potato leaves, dandelion leaves, and spinach leaves [5]. The carotenoid content of plants depends on various factors, including genetic predisposition, state of ripeness, environmental conditions (e.g., increased temperature and light availability contribute to increased carotenogenesis in fruit), and cultivation method. Inappropriate post-harvest storage, warehousing, or processing may result in losses in carotenoid content. The main cause is enzymatic and non-enzymatic oxidation due to light, heat, the presence of metals, enzymes, and peroxides, and an acidic or alkaline environment. To prevent the loss of carotenoids, they can be protected by adding antioxidants or neutralizing agents [6,7].

Due to their wide spectrum of properties, carotenoids are used in the food, pharmaceutical, feed and cosmetic industries. Some carotenoids (e.g., beta-carotene, lutein, zeaxanthin or lycopene) are produced industrially on a large scale and used as food or supplement ingredients [4]. The carotenoid market is estimated to grow from USD 1.5 billion in 2019 to USD 2.0 billion in 2026 as a result of the growing interest in the use of natural carotenoids as food colorings and due to innovations in carotenoid extraction [8].

### 1.2. Extraction Process

Extraction is the process aimed at the physical separation of components of a mixture based on differences in their solubility in two immiscible liquids or their affinity for an absorbent. There are two most common extractions: solvent extraction and solid-phase extraction. Solvent extraction is based on extracting non-polar, uncharged particles in an aqueous system into an immiscible organic solvent or on extracting polar, ionized particles from an organic solvent into an aqueous solution. Solid-phase extraction involves passing a sample solution through a sorbent layer so that the analyte is retained and matrix constituents are eluted, or vice versa [9]. The extraction efficiency depends primarily on the properties of the sample from which the analyte is to be extracted. The extraction process consists of several main steps. The first step is the desorption of the compound from its place in the matrix. Then, the compound is diffused through the organic part of the matrix to reach the boundary between the matrix and the liquid. At this stage, the compound reaches the extraction phase. The final step is the collection of the extracted analyte [10].

The most common extraction method for carotenoids is the solvent extraction method using petrochemical solvents. Solvent extraction of carotenoids from vegetables is much more difficult than raw materials, such as fats, meat, or vitamin supplements, because of the process conditions that may contribute to the degradation of the compounds. The extracted substance must be well soluble in the extractant used. The choice of solvent should follow the principle that “similar dissolves in similar”. In the case of carotenoids, the most common organic solvents are chloroform, hexane, isopropanol, methylene chloride, or diethyl ether, which unfortunately pose environmental (water and air), health (acute and chronic toxicity) and safety (explosion and decomposition) risks. The decreasing polarity of the solvent allows the extraction of different compounds. The advantage of solvent extraction is the lack of specialized apparatus and the simplicity of execution [2]. However, the disadvantages of the method include prolonged exposure time, the generation of hazardous volatile organic compounds, low process efficiency, the necessity to have separate evaporators, reagent residues after evaporation, and, above all, the harmful effects of the method on the environment. Nowadays, solvents of petrochemical origin, including n-hexane, which is the most common solvent in carotenoid extraction, are strictly regulated by European directives. Agents are subject to registration, evaluation, authorization and restriction of chemicals (REACH) [11].

### 1.3. Extraction and Sustainable Food Production

Thus, green extraction methods are gaining importance, characterized by fast extraction rates, minimal thermal effects on the extracted compounds, no hazardous volatile residues, and lower water consumption and wastewater production [12]. Green extraction methods use only safe and non-toxic solvents produced from renewable biomass sources, such as starch, wood, and vegetable oils or environmentally friendly petrochemical solvents, non-toxic and/or biodegradable. Such solvents include, but are not limited to: 2-methyl-hydro furan (2-MeTHF); ethyl acetate; isopropanol; dimethyl carbonate (DMC); cyclopentyl methyl ether (CPME); and ethyl lactate [2,11,13]. 

An alternative to the use of green solvents in the extraction process are deep eutetic solvents (DES). They are referred to as the new generation of iconic liquids (ILs). Both groups of solvents are characterized by the same physical properties, with completely different chemical properties. The main advantages of DES, compared to ILs, are the ease of preparation and the easy availability of relatively inexpensive ingredients [14]. DES are prepared by mixing a hydrogen bond acceptor (HBA) with a hydrogen bond donor (HBD). They represent a mixture of asymmetric ions that have low crosslinking energies and thus low melting points. The general notation of DES compounds can be represented by the formula Cat^+^X^−^zY, where Cat^+^ represents any phosphonium or sulfonium ammonium cation and X represents a Lewis base (usually a halide anion). Between X^−^ and the Lewis or Brønsted acid Y, complex anionic forms are formed, where “z” refers to the number of molecules of Y that interact with the anion [14]. The first DES compounds were synthesised using choline chloride and urea. Subsequently, compounds, such as carboxylic acids (e.g., succinic acid, phenylacetic acid and citric acid) or glycerol, were introduced. In the year 2011, the possibility of using plant metabolites for the synthesis of DES, such as amino acids, sugars, or organic acids (melanic acid, malic acid and aconitic acid), was discovered, which were termed natural deep eutetic solvents (NADESs) [15]. NADESs are specific solvents, thus it is possible to adjust their properties in such a way so as to obtain a high extraction efficiency of poorly water-soluble compounds. This makes NADESs a promising solution for the extraction of beta-carotenoids. The process uses non-toxic, biodegradable solvents that do not affect the extracted extractant. As a result, the purification step of the extract can be omitted, and the extract can be directly used in food [16].

The topic of sustainable development was already taken up in 1992, at the initiative of the U.N., at a conference in Rio de Janeiro. As a result, a document called “Agenda 21” was developed and approved, including a program to implement sustainable development systems in local life. The impetus for action was the observed changes in the environment under the influence of increasing globalization and industrialization [17]. In 2015, in New York, the Sustainable Development Goals, included in the 2030 Agenda, were prepared and unanimously adopted by all United Nations member states. The document contains 17 items, each underpinned by specific tasks to be achieved by 2030. For example, goal 12 relates to sustainable food consumption and production. It includes such tasks as halving the global amount of wasted food per capita, retail sales and consumption, and reducing food losses in production and distribution. Another important subpoint is to ensure environmentally sound management of chemicals and all types of waste throughout their life-cycle, in line with established international frameworks, and to significantly reduce the level of release of these substances into the air, water and soil, thereby minimizing their negative impact on human health and the environment [18].

The subitems mentioned above constitute only a part of the tasks related to objective 12, “Responsible Consumption and Production”. Nevertheless, the recovery of carotenoids from the byproducts of fruit and vegetable processing using green extraction methods is fully in line with Agenda 2030, addressing the problem of food waste and reducing the use of reagents that are harmful to the external environment.

### 1.4. Utilization of Byproducts in Carotenoid Extraction

Progressive globalization and increased industrialization contribute to a higher food production. Nevertheless, the lack of adequate management and infrastructure has contributed to significant losses and wastage of finished products, raw materials, and byproducts [19]. Currently, food waste is one of the main problems worldwide. According to 2011 data from the Food and Agriculture Organization of the United Nations (FAO), approximately 30% of the food produced in the world that is still suitable for consumption is thrown away each year, i.e., about 1.3 billion tons of food. Food loss occurs at every stage of the supply chain, including food harvesting, transportation to packing houses or markets, grading, storage, marketing, processing, and homes before or after cooking [19]. According to the 2016 food loss index, from harvest to distribution, on average, around 13.8% of food is wasted worldwide [20]. The greatest losses are in fruit and vegetables due to their perishable nature. More than 20% of the world’s fruit and vegetable production is lost after harvesting. In addition, the processing of the vegetables and fruits that have been harvested regenerates significant amounts of byproducts in the form of non-edible parts, such as peels, cores and pomace, but also unripe or damaged vegetables and fruits, which continue to be a source of biologically active compounds [21]. Plants accumulate bioactive compounds in both edible and non-edible parts. Carotenoids constitute one of the largest groups of pigments, ranging from yellow to red. They are stored largely in the skins of vegetables and fruits, e.g., tomatoes, oranges, grapes, and carrots, that are often discarded. The production of juices, for example, generates about 5.5 million metric tons of byproducts, and the production of canned and frozen foods generates up to 6 million metric tons of vegetable waste [19]. During carrot juice production alone, up to 50% of the weight of the raw material is lost, which still has a high carotenoid content in the form of pomace, but is not further processed for economic and logistical reasons [22].

**Figure 2:**
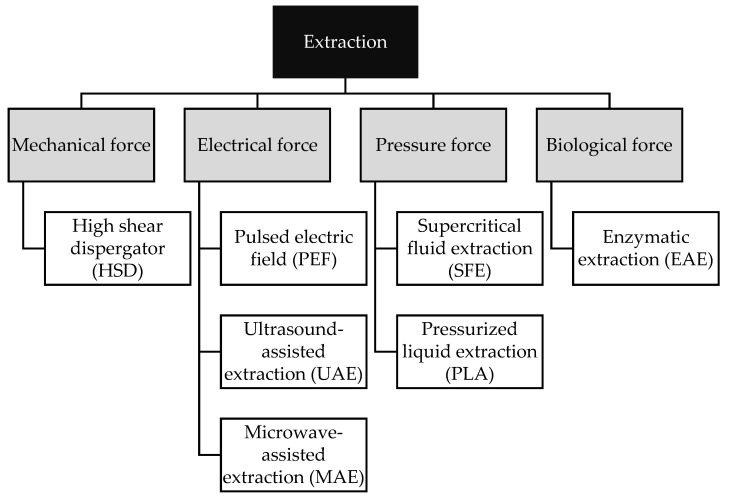
Scheme of the green extraction methods.

The following article presents solvent-free green extraction methods based on environmentally safe reagents instead of the traditionally used petrochemical solvents (Figure 2). The lipophilic nature of carotenoids allows the use of vegetable oils as a solvent that increases the solubility of biologically active compounds while not contributing to their degradation [12]. The solvent-free extraction process can be supported by high shear dispersive extraction, pressurized liquid extraction, microwave extraction, ultrasonic extraction, electric field extraction, supercritical extraction and enzyme-assisted extraction. A detailed discussion of the methods is given later in this article (Table 1).

## 2. Extraction Using a High Shear Dispersant (HSD)

Using a high shear dispersant in the extraction process allows the mechanical disruption of the cell wall and membranes, facilitating the release of compounds confined within the cell. The technique is much less time consuming than the traditional carotenoid extraction method. The use of edible vegetable oils in the extraction of carotenoids not only provides an environmentally friendly alternative to organic solvents, reduces the energy consumption of the extraction process and makes it possible to obtain an uncontaminated carotenoid extract ready for direct use without purification. Vegetable oils are biodegradable, nontoxic and perform similarly to petrochemical solvents. An obstacle to the use of vegetable oils is their high viscosity, which strongly reduces solvent diffusivity, even at elevated temperatures [23,24]. Edible oils are characterised by a significant amount of polyunsaturated bonds and are thus more likely to be oxidised. The enrichment of oils with antioxidant compounds, such as carotenoids, extends the shelf life of the product without the need for synthetic antioxidants [25].The findings presented in Tiwari et al. (2019) and Baria et al. (2019) indicate that the method is more effective in extracting carotenoids even than ultrasonic-assisted extraction.

Tiwari et al. (2019) optimized the biorefinery of carotenoids from carrot pomace using linseed oil. First, carrot pomace was enzymatically pretreated to increase the availability of carotenoids in the cells. For this purpose, samples were blanched and then treated with cellulase and pectinase. The use of a prior enzyme treatment increased the amount of extracted carotenoids from 53.86 ± 0.0084 μg/g to 73.03 ± 1.182 μg/g. The extraction was carried out with a high shear disperser (HSD); the following parameters were used: 20,000 rpm, 12 min, solvent to expeller ratio was 1:1. As a result, 82.66 ± 0.06 μg/g of carotenoids were obtained [22].

Baria et al. (2019) developed a method to extract carotenoids from enzyme-treated mango pulp (pectinase and cellulase). In the extraction process using a high shear dispersant, three types of vegetable oils were employed—peanut, sunflower and linseed. Linseed oil proved to be the most beneficial oil. In turn, the most optimal choice of method parameters was determined for 20,000 rpm, 4 min, and a linseed-oil-to-mango-pulp ratio of 2:1. As a result, 21.77 ± 0.09 μg/mL of carotenoids were obtained [26].

## 3. Pressurized Liquid Extraction (PLE)

Pressurized liquid extraction (PLE) is also called accelerated solvent extraction (ASE). The method involves extraction using liquid solvents at an elevated temperature and pressure, which accelerates the extraction process by facilitating cell permeability. Simple alcohols (ethanol and methanol) or their mixtures with water are recommended as solvents for green extraction methods [2,10]. In order to avoid the use of organic solvents, extraction using pressurized liquids has gained popularity, with studies proving that the efficiency of the method is comparable to that obtained using conventional extraction methods. The technique is relatively similar to Soxhlet extraction, but the high pressure keeps the solvent below the boiling point, thus protecting thermolabile compounds from degradation. In addition, it increases the solvent permeability and the availability of the biologically active components, resulting in reduced solvent consumption [3]. There are two methods of conducting the process—dynamic and static. The first one consists of a continuous supply of solvent through pumps. The static pressurized liquid extraction method consists of one or more extraction cycles with a solvent exchange between cycles. Regardless of the method chosen, a wide range of extraction temperatures (20–200 °C) and pressures (35–200 Bar) can be applied. In the static extraction method, temperature and process time play a key role, while the extraction efficiency depends on the solubility of the analyte in the solvent [10]. The main advantage of pressurized liquid extraction is the much faster process time and the much lower solvent usage. Moreover, the method can be more efficient for polar compounds than supercritical fluid extraction. However, the limitation of the method is the content of analytes in the tested sample, as the maximum sample mass limit is 10 g. An additional limitation may be the cost of the equipment necessary for the process [19].

Cardenas-Toro et al. (2015) proposed the extraction of carotenoids from compressed palm fiber by pressurized liquid extraction with heated ethanol. The study showed a positive effect of temperature on carotenoid recovery. The effect of three temperature levels was investigated: 35 °C, 45 °C, and 55 °C. It was observed that a better performance was obtained at 35 °C and 55 °C, where the carotenoid recovery was similar and higher than at 45 °C. This could be because, at 45 °C, the rapid degradation of carotenoids can occur, while at 55 °C, the rate of solvent penetration and diffusion of bioactive compounds is much faster than their degradation. A temperature of 35 °C and a pressure of 4 MPa were chosen as optimal process conditions, which were characterized by high yields with low energy consumption [27].

Šaponjac et al. 2021 proposed an optimised method for the high-pressure-assisted extraction of carotenoids from carrots. Carrots were crushed, dried by freeze drying and ground. The optimum conditions were determined at 80 °C, 5 min, S/L 1:4, 10.34 MPa. A mixture of acetone (25%) and ethanol (75%) was used as solvent. The extraction resulted in 27 mg of total carotenoids per 100 g of extracted raw material [28].

## 4. Microwave-Assisted Extraction (MAE)

The microwave-assisted extraction process (MAE) is based on the phenomenon of radiation absorption by particles of the substance, from which biologically active compounds are extracted. The use of microwaves in the extraction process greatly simplifies extracting the active compound. The method is commonly used to extract selected compounds from solids. The process uses the phenomenon of radiation absorption by the substance particles. The technique is economical as it reduces the extraction time and allows less solvent consumption. This is due to the way the heat energy is transferred. Traditional heating by convection or conduction takes much longer than using microwaves. Microwave heating is based on the movement of dipole particles, which produce heat energy through friction. Due to using a non-polar solvent that does not absorb microwave radiation, the sample is heated and releases the heat to the extractant. Unfortunately, increasing the extraction time may contribute to the thermal degradation of carotenoids. In order to increase the efficiency of carotenoid extraction from carrots, they can be subjected to blanching with the addition of ascorbic acid. Studies have shown that the recovery of carotenoids and their antioxidant activity was significantly higher than in the absence of the prior treatment. In addition, the blanching process in an acidic environment contributed to the softening of the raw material structure by the solubilizing pectin, which increased the availability of carotenoids [2].

Chutia and Mahanta (2021) optimized a method for extracting carotenoids, using olive oil, from passion fruit peels using microwaves by applying 200 W of microwave power, a process duration of 25 min, and a raw-material-to-solvent ratio of 10 g / 100 mL. The extraction yielded 1178.54 μg of carotenoids from 100 g of passion fruit peels. Before the extraction process, the raw material was freeze dried and then ground to obtain a homogeneous powder [12].

Baria et al. (2019) tested microwave-assisted extraction using three types of oils, peanut, sunflower and linseed, for the extraction of carotenoids from mango pulp. The material studied underwent a prior enzymatic treatment, using pectinase and cellulase. In the results obtained, the publication authors point out the low efficiency of the process with the para meters used: 100 W, and times of 2, 4, 6, and 8 min. The low efficiency of the process may be due to the low value of polar groups in the mango pulp, which leads to reduced microwave penetration in the food matrix. In addition, an increase in temperature due to microwave penetration was observed during the experiment, which could further contribute to a decrease in the carotenoid content of the raw material tested [26].

Elik, Yanik, and Göğüş (2020) investigated the extraction of carotenoids using linseed oil from carrot pomace produced after juice production. The pomace was dried using lyophilization and then ground. By optimizing the method, using the parameters of 165 W, 9.39 min and an oil-to-carrot-pomace ratio of 8.06:1, the percentage of carotenoid recovery was 77.48% [29].

Sharma et al. (2022) performed MAE extraction using extra virgin olive oil and refined corn oil from sea buckthorn pomace. The pomace was freeze dried, the seeds were manually removed and then milled. The optimized parameters used were 130 W, 30 min, and a sample-to-solvent ratio 1:10. Based on the results, olive oil appeared to be the better solvent. The carotenoid extraction was 28.3 mg/100 g of oil extract; in the case of maize oil, the content of extracted carotenoids was 26.91 mg/100 g of oil extract [30].

## 5. Ultrasound-Assisted Extraction (UAE)

The use of ultrasound in the extraction process can significantly improve the efficiency of the process, lower the temperature necessary for the process to take place and shorten the extraction time, thus enabling the better preservation of the properties of the biologically active compounds [31]. The use of vegetable edible oils in carotenoid extraction not only provides an environmentally friendly alternative to organic solvents, but additionally acts as an oxygen barrier and significantly delays the oxidation and degradation of bioactive compounds. In addition, the method provides a solution to the problem associated with the high viscosity of edible oils, which reduces solvent diffusivity, even at elevated temperatures. The use of ultrasound significantly increases the extraction efficiency, without the need to heat the medium [23]. The method can be applied to solid or semisolid samples. The ultrasound-assisted extraction process is based on the phenomenon of cavitation, i.e., the rapid conversion of a liquid phase to a gas phase under reduced pressure [32,33]. Cavitation is the formation, growth and collapse of microbubbles within a liquid subjected to high-frequency sound waves (> 20 kHz). These transformations result in a violent collision of molecules, which creates shock waves, thus creating regions of very high temperature (5500 °C) and pressure (up to 50 MPa) for a short time (9–10 s) [34]. Damage to the cell wall allows better solvent penetration and the leaching of intracellular carotenoids. The effective disintegration of the cell wall can increase the efficiency of the process up to tenfold. A major advantage of ultrasound-assisted extraction is that it can be performed at room temperature, which allows the thermolabile properties of the analytes to be preserved [2,31]. The main disadvantages of the method are the sudden temperature changes caused by the rapid cavitation phenomenon, which can cause undesirable reactions, such as thermo-oxidation and volatilization of low-volatility compounds. In order to prevent this phenomenon, the performance of the cavitation agents should be optimized. These factors include force, frequency, sonication density, probe type, solvent/substrate ratio, solid material properties, external temperature and pressure, and extraction time [35].

Tiwari et al. (2019), in their study, presented the extraction of carotenoids using ultrasound from carrot pomace. Carrot pomace was previously subjected to enzymatic treatment using cellulase and pectinase. The material prepared in this way was then subjected to ultrasound extraction in the presence of linseed oil. The result obtained was no less than that from the extraction process using a high shear dispersant (HSD). The following parameters were used for the UAE process: duty cycle 45%, probe radius 13 mm and 750 W, 12 min and a solution-to-pulp ratio of 1:1. The result was 21.67 ± 0.40 μg/g carotenoids [22].

Civan and Kumcuoglu (2019), in their study, presented an optimized method to extract carotenoids and capsaicin from red Jalapeño peppers using olive oil. The raw material was separated from the seeds, then cooked and prepared into a homogeneous paste. The resulting pulp was previously dried and ground. UAE parameters were optimized to the following: 60% amplitude, 60 °C, and time 5 min. The experiment yielded 230.54 mg beta-carotene/100 g product [35].

Chutia and Mahanta (2021), in their study, presented a method to extract carotenoids, using olive oil, from passion fruit peel using ultrasound. Before the extraction process, the raw material was freeze dried and then ground to obtain a homogeneous powder. The optimized method was based on olive oil, with the following parameters: 100 W, 46.59 °C, 39.06 min and a solid to solvent ratio was 29.9 g/100 mL. The process yielded 1241.95 μg from 100 g of dried passion fruit peels [12].

Bhimjiyani et al. (2021) optimized an ultrasound-assisted extraction method of carotenoids from sea buckthorn pomace using linseed oil. The pomace was previously dried in a solar dryer. It was observed that the use of ultrasound significantly improved the efficiency of the extraction process by up to 50%. The following parameters were considered optimal process conditions: 75.5 min, 80.8%, frequency 20 kHz and dosage 19.9. The procedure resulted in 14.2 mg/L of carotenoids recovered from the supernatant [36].

Stupar et al. (2021) in their study presented an ultrasound-assisted extraction of carotenoids from pumpkin using natural deep euthetic solvents. The raw material under study was crushed and then dried by freeze drying. To prepare ten different NADESs, DL-menthol, octanoic acid, nonanoic acid, decanoic acid and dodecanoic acid were mixed in appropriate proportions. As a result of the experiment, the best solvent was found to be C8:C10 (3:1), which was used in the extraction using UAE. The optimised process conditions were determined to be 50 °C, 52.5 W/cm^3^, S/L 7 mL/g and 10 min. UAE-assisted extraction resulted in 151.41 μg/mL of beta-carotene, in comparison with extraction using NADESs alone, which has a yield of 96.74 1 ± 0.03 μg/mL [16].

## 6. Pulsed Electric Field Assisted Extraction (PEF)

Applying an electric field in the extraction process can significantly improve the yield and lower the temperature, which is particularly important for thermolabile compounds. Electric field extraction involves the application of strong external electric fields (1–50 kV/cm), for short periods (microseconds to milliseconds), to cellular material, inducing the electroporation of cell membranes. The electrical potential passes through the cell membrane and separates molecules according to their charge [37]. This repulsion electroporation causes reversible or irreversible pore formation in cell membranes, thereby increasing the permeability of the cell membrane for ion and macromolecule transport. The method’s main advantages include the absence of high temperatures involved in the process, low energy consumption, high efficiency and low process costs.

Additionally, the method is environmentally friendly due to the lack of petrochemical solvents. Electric field extraction is characterized by the high quality and purity of the extracts. The method uses renewable plant materials and alternative solvents, such as water or agro-solvents (ethanol and fatty acid methyl esters from plant oils). Additionally, the method improves the selective extraction of biologically active ingredients without destroying the matrix [37]. The disadvantages of the method include the need to adapt it to each type of sample, as the process parameters are dependent on the electrical conductivity and texture of the raw material [2,37,38,39].

López-Gámez et al. (2021) proposed the electric-field-assisted extraction of carotenoids and polyphenols from carrot purée using olive oil. They observed that thermal pretreatment was less effective in extracting biologically active components than electro-permeabilization, especially polyphenols. The results obtained indicate the effectiveness of PEF application in increasing the bioavailability of biologically active components, with no loss of carotenoids and without adverse changes in the raw material. The proposed process conditions were 5 pulses of 3.5 kV/cm and frequency of 0.1 Hz [39].

Pataro et al. (2020) studied the extraction of lycopene from tomato pomace using an electric field. The experiment used two solvents: acetone, a popular compound used in carotenoid extraction, and ethyl lactate, which has a low environmental impact. As a result of hydrolysis, it dissociates into safe compounds: lactic acid and ethanol. It was observed that the most important parameter in the process was the extraction time used; the most optimal time was determined to be 240 min. The application of an electric field in the extraction process significantly improved the efficiency of the process. Additionally, in the case of ethyl lactate, higher amounts of all-trans lycopene of about 23% (for acetone, it was 18%) were observed, whose presence stabilizes and intensifies the color of the extract. The process resulted in 6311 ± 254 mg of lycopene per kilogram of tomato peels [13]. 

## 7. Supercritical Fluid Extraction (SFE)

Supercritical extraction is an advanced carotenoid extraction technology that is at the same time environmentally friendly. A supercritical fluid can be defined as any substance at a temperature and boiling point above its critical point. Supercritical fluid techniques have better properties than conventional methods using organic solvents. The most commonly used supercritical fluid is supercritical CO_2_, which is cheap, chemically inert, flammable, readily available in high purity and recyclable. The main advantages of using supercritical CO_2_ include low temperature and low pressure, which are important for extracting natural substances, especially thermally unstable components. As the substance approaches the critical temperature, the properties of the gas and liquid phases converge. This process results in a single phase that does not differentiate between liquid and gas phases, and the heat of vaporization is zero at the critical point and beyond [3,40]. For CO_2_, the critical point is at 31.1 °C (304.2 K) and 7.3 MPa (72.8 bar), making it possible to work at room temperature and mild pressure, which is ideal for the extraction of thermolabile compounds [41].

Furthermore, supercritical fluids are characterized. This makes them excellent for extracting natural compounds, including those not yet described. In addition, the carotenoid extracts obtained are characterized by high concentration and purity, with no residual solvent in the final product [3,40].

The extraction of carotenoids using SC-CO_2_ alone gives a relatively low extraction rate of about 34% due to the high molecular weight of the extracted compounds. The use of ethanol as a solvent increases the recovery of carotenoids. Ethyl alcohol potentiates the polarity of CO_2_ by dissolving macronutrients, such as carbohydrates, proteins, and lipids. In addition to the use of solvent in the process, the pressure and temperature used are also very important and can greatly contribute to the efficiency of the process. High pressure can disintegrate cell walls, thus increasing their availability during the extraction process. Similarly, the elevated temperatures can increase the extraction of bioactive components, but excessive temperatures can have the opposite effect, degrading and isomerizing carotenoids. The most optimal process conditions are a pressure between 200–450 Ba and a temperature between 50–70 °C [41].

De Andrade Lima et al. (2018) optimized a method for extracting carotenoids from carrot peels, using CO_2_, in supercritical conditions. The raw material was previously freeze dried and then ground. The optimized extraction conditions allowed a carotenoid recovery of 96.2%, with a total extraction of 9.89 mg of carotenoids [42].

De Andrade Lima et al. (2019) designed an experiment to recover carotenoids from vegetable byproducts using supercritical CO_2_. The study evaluated the feasibility of extracting carotenoids from raw materials, such as sweet potato pulp, sweet potato peels, tomato pulp, tomato peels, apricot pulp, apricot peels, pumpkin pulp, pumpkin peels, peach pulp, peach peels, green pepper pulp, yellow pepper pulp, red pepper pulp, pepper waste and a mixed sample of different wastes. An optimized method was used in the experiment, which included the following conditions: S-CO_2_ use at 15 g/min, 59 °C, 350 bar, 15.5% (*v*/*v*) ethanol, 30 min. The results obtained indicate that the method was highly optimized. For all types of raw material, the recovery of total carotenoids was between 91.0% and 99.8%. The highest percentage extraction level was observed for peach flesh, while the lowest was for tomato peel. Quantitatively, the total carotenoids extracted from the sweet potato flesh were about 430.6 ± 27.7 μg/g [43].

**Table 1:** Summary of extraction methods.

	Raw Material	Extraction Medium	Optimized Conditions	Results mg/100 g	References
**Extraction with High Shear Dispergator (HSD)**					
1.	Carrot biowaste	Flaxseed oil	20,000 rpm, 12 min, S/L 1:1	8.27	[22]
2.	Mango pulp	Flaxseed oil	20,000 rpm, 4 min, S/L 2:1	2.18	[26]
**Pressurized Liquid Extraction (PLA)**					
3.	Pressed palm fibre	Hot Ethanol	4 MPa, 35 °C, 17 min, flow rate 2.4 g/min	4400	[27]
4.	Carrot	Aceton: Ethanol	10.34 MPA, 80 °C, 5 min, S/L 1:4	27	[28]
**Microwave-assisted Extraction (MAE)**					
5.	Passion fruit peels	Olive oil	200 W, 25 min, S/L 1:10	1.18	[12]
6.	Mango pulp	Flaxseed oil	100 W, 6 min, S/L 1:2	1.07	[26]
7.	Carrot byproducts	Flaxseed oil	165 W, 9.9 min, S/L 1:8.06	413.28	[29]
8.	Seabuckthorn pomace	Olive oil	130 W, 30 min, S/L 1:10	28.3	[30]
**Ultrasound-assisted Extraction (UAE)**					
9.	Carrot biowaste	Flaxseed oil	0.45 duty cycle, 13 mm probe radius, 750 W, 12 min, S/L 1:1	2.17	[22]
10.	Red Jalapeño pepper	Olive oil	0.4 duty cycle, 400 W, 24 kHz, 60 °C, 5 min, S:L 0.4 g/mL	230.54	[35]
11.	Passion fruit peels	Olive oil	100 W, 46.59 °C, 39.06 min, S/L 29.9 g: 100 mL	1.24	[12]
12.	Seabuckthorn pomace	Flaxseed oil	Amplitude 80%, 20 kHz, 75.5 min	1.42	[36]
13.	Pumpkin	NADES	50 °C, 52.5 W/cm^3^, S/L 1:7, 10 min	15.14	[16]
**Pulsed Electric Field Assisted Extraction (PEF)**					
14.	Carrot puree	Olive oil	5 impulse, 3.5 kV/cm, 0.1 Hz	21.5	[39]
15.	Tomato byproducts	Ethyl lactate	5 kJ/kg, 5 kV/cm, 240 min	631.1	[13]
**Supercritical Fluid Extraction (SFE)**					
16.	Carrot biowaste	CO_2_	59 °C, 349 bar, 15.5% ethanol,	9.89	[42]
17.	Vegetable biowaste	CO_2_	15 g/ min, 59 °C, 350 bar, 15.5% ethanol, 30 min	43.06	[43]
**Enzyme-assisted Extraction (EAE)**					
18.	Sweet peppers	-	Viscozyme L, pectinase	41.37	[44]

## 8. Enzyme-Assisted Extraction (EAE)

Enzymes act as ideal catalysts that can assist in the extraction of various biologically active compounds of natural origin. The use of enzymes enables the degradation or disruption of cell walls and membranes, thereby increasing the availability of bioactive components. Compared to microwave or ultrasound-assisted extraction, enzyme-assisted extraction has better processing parameters. It is a higher yielding method with lower energy expenditure. The most commonly used enzymes in the method are proteases, pectinases, cellulases, tannases and carbohydrates. Enzyme-assisted extraction is characterized by high efficiency, rapidity and selectivity for biologically active components. It is safe for thermolabile compounds and ensures high purity of carotenoids. Additionally, the method is safe for the external environment. As a result, the enzyme technique has been increasingly used to extract bioactive compounds from plants [41]. Disadvantages of the method include the high cost of enzymes, relative to volume, and the high dependence of enzymes on pH and temperature [45].

Nath et al. (2016) optimized the extraction of carotenoids using enzymes from sweet peppers. The raw material was washed, sliced, and blanched to prevent auto-oxidation and soften the material, contributing to higher carotenoid availability. The effect of three enzymes, viscozyme L, pectinase, and cellulase, which have liquefaction capacity, was investigated to recover the total carotenoids. It was observed that viscozyme L and pectinase had a higher liquefaction capacity than cellulase, as evidenced by a higher extraction efficiency (80–87%). The best results were obtained with 0.3% viscozyme and pectinase enzymes. The recovery of carotenoids after enzymes increased about 2.3 times compared to the standard method using n-hexane [44].

## 9. Saponification

One of the processes that increases the efficiency of carotenoid extraction is saponification. In most cases, carotenoids occur in free form, but xanthophylls can also occur in free form or as esters of higher carboxylic acids, such as lauric, stearic, linoleic or oleic acid. The saponification process mainly removes esterified xanthophylls, chlorophylls, and lipids to not interfere with the chromatographic analysis. The saponification process is unnecessary for materials with a low lipid content, which do not contain xanthophyll esters (e.g., leafy vegetables). The saponification process is often carried out separately after the extraction process. Running the processes in parallel may contribute to a reduction in extraction efficiency.

The efficiency of saponification, particularly the amount of qualitative and quantitative losses of carotenoids, depends largely on the conditions of the process. Therefore, the applied temperature, the potassium hydroxide (KOH) concentration, the hydrolysis time, and the volume used for partition and washing are of great importance. There are two methods of saponification of free carotenoids—hot (56 °C, 20 min) and cold (25 °C, 16 h). Both methods have strengths and weaknesses. Cold saponification is very time consuming, while high temperatures in the process contribute to the isomerization and degradation of carotenoids [2].

## 10. Conclusions

Applying green extraction methods to extract carotenoids from the byproducts of fruit and vegetable processing responds to market demands. Furthermore, it is in line with the global goals of Agenda 2030 related to the sustainability of food systems. The use of non-edible and wasteful plant parts can make a significant contribution to reducing food waste. On the other hand, green extraction methods are environmentally safe and highly efficient. In addition, green extraction methods are characterized by a shorter extraction time of biologically active compounds, which lowers energy consumption. Additionally, they do not require the use of petrochemical solvents, which significantly reduces the harmfulness of the process for the environment and solves the problem of the subsequent disposal of toxic waste.

## Data Availability

All details are reported in the manuscript.
